# New Insights into Pathogenic Vibrios Affecting Bivalves in Hatcheries: Present and Future Prospects

**DOI:** 10.3389/fmicb.2017.00762

**Published:** 2017-05-03

**Authors:** Javier Dubert, Juan L. Barja, Jesús L. Romalde

**Affiliations:** Departamento de Microbiología y Parasitología, CIBUS-Facultad de Biología, Universidade de Santiago de CompostelaSantiago de Compostela, Spain

**Keywords:** *Vibrio*, bivalve hatchery, pathogenesis, antibiosis, probiosis, phage therapy

## Abstract

Hatcheries constitute nowadays the only viable solution to support the husbandry of bivalve molluscs due to the depletion and/or overexploitation of their natural beds. Hatchery activities include the broodstock conditioning and spawning, rearing larvae and spat, and the production of microalgae to feed all stages of the production cycle. However, outbreaks of disease continue to be the main bottleneck for successful larval and spat production, most of them caused by different representatives of the genus *Vibrio*. Therefore, attention must be paid on preventive and management measures that allow the control of such undesirable bacterial populations. The present review provides an updated picture of the recently characterized *Vibrio* species associated with disease of bivalve molluscs during early stages of development, including the controversial taxonomic affiliation of some of them and relevant advances in the knowledge of their virulence determinants. The problematic use of antibiotics, as well as its eco-friendly alternatives are also critically discussed.

## Introduction

According to latest SOFIA report (FAO, 2016), the worldwide food production must be increased considerably since global population will reach 9.7 billion people in 2050. In this context, marine products are an essential part of the human diet as one of the main resources of animal protein and its worldwide consumption *per capita* has been duplicated since 1960. Nowadays, more than half of these products come from aquaculture due to the overexploitation of the traditional fisheries and this proportion will exceed 65% in 2030. Bivalves are one of the most important food products for the aquaculture industry and the worldwide production (mainly oysters, mussels, clams and scallops) was close to 14 Mt with an economic value of more than $16 billion (FishStatJ, FAO).

**FIGURE 1:**
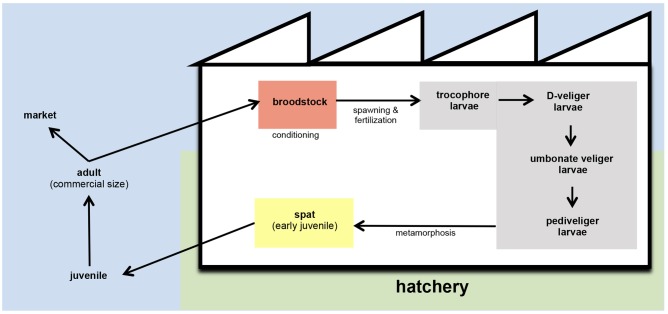
**Schematic representation of bivalve husbandry**. In hatcheries, broodstock are kept in conditioning tanks (red) until spawning. After fertilization, embryos are transferred to larval tanks (gray) where larvae reach the different stages of development. From settlement and metamorphosis, spat is kept in spat tanks (yellow) and grow until the adequate size to carry on the culture out of the hatchery. In the field, juveniles are fattened with the nutrients content in natural environment until they reach the commercial size.

Depletion and/or overexploitation of natural beds promoted that bivalve hatcheries gained importance in the shellfish aquaculture as the only viable solution to support the bivalve husbandry (Ojea et al., 2008; da Costa et al., 2013). Hatcheries generally provide spat of different bivalve species to the shellfish farmers (**Figure 1**). The term spat is applied to the early juvenile stage of bivalve development. It is commonly applied to juveniles in hatcheries and is related to bivalve larvae that have set and undergone metamorphosis. Then, spat is fattened in the natural environment until reach the commercial size (Helm and Bourne, 2004). These authors also described the term seed as the juvenile products supplied by hatcheries to shellfish farmers. In other cases, these facilities could also supply mature larvae to the farmers, as occurs with pediveliger Pacific oyster (*Crassostrea gigas*) larvae on the Pacific coast of North America (Helm and Bourne, 2004).

**FIGURE 2:**
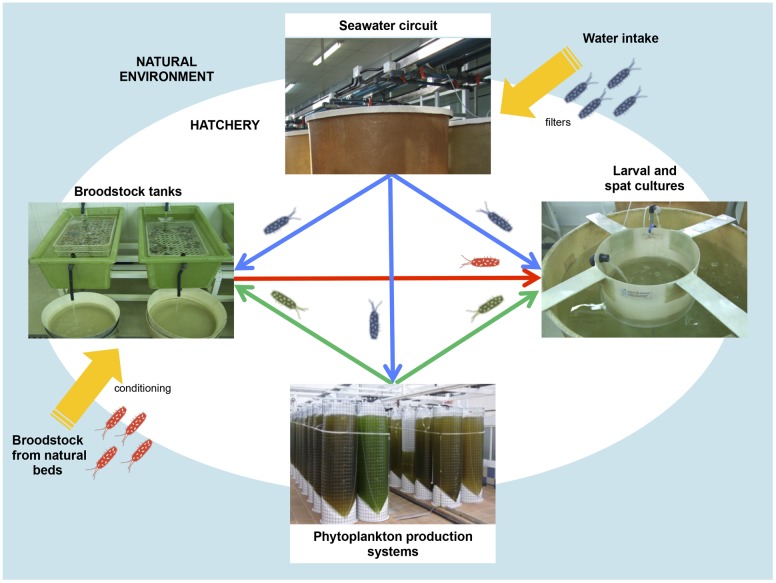
**Bacterial feedback among different hatchery compartments: broodstock conditioning tanks, phytoplankton production system, seawater circuit and larval and spat tank**.

Hatchery activities include the broodstock conditioning and spawning, rearing and setting larvae, rearing spat to an acceptable size and the production of large quantities of microalgae to feed all stages of the production cycle (**Figures 1**, **2**) (Prado et al., 2010). According to the **Figure 1**, the first step in hatchery culture is the broodstock conditioning which is performed in tanks where maturation of individuals is induced by artificial means until spawning. After fertilization, embryos are transferred to larval culture tanks where larvae reach the different stages of development. Bivalve cultures continue in spat tanks from settlement and metamorphosis, where spat is kept until they reach the adequate size to be transfer to the natural environment (Helm and Bourne, 2004; Dubert, 2015; Dubert et al., 2016a). All these hatchery activities are highly susceptible to bacterial contamination (e.g., seawater and broodstocks are introduced from the natural environment, phytoplankton cultures are not axenic, improper management of the cultures and seawater circuit…) or cross contamination as consequence of the bacterial feedback among compartments (vertical transmission from broodstocks to larvae, through the phytoplankton used as food…) (**Figure 2**) (Dubert, 2015; Dubert et al., 2015, 2016a). Special attention should be focused on seawater, the common nexus among different compartments, which is generally renewed in larval and spat tanks every 2 days after filtration and UV-sterilization. In addition, and due to their filter feeding nature, bivalves act as a bacterial reservoir, including vibrios, and can release them to the seawater even after every renewal (Prado et al., 2014a). Broodstock, phytoplankton or seawater are key players in the *Vibrio* dissemination within the hatchery, especially to larval and spat cultures (Beaz-Hidalgo et al., 2010; Prado et al., 2014a; Romalde et al., 2014; Dubert et al., 2015, 2016a; Holbach et al., 2015). Interestingly, microbiota reported in some of these studies included opportunistic pathogens harmless to broodstock or microalgae, but potentially harmful to larvae or spat. In summary, this bacterial feedback among the different hatchery compartments seems to be inevitable and its correct management plays an essential role in the successful working of the shellfish hatcheries (Dubert, 2015; **Figure 2**).

Clearly, microbiological aspects play an important role in the successful bivalve culture. Knowledge of *Vibrio* populations is essential since vibriosis caused by pathogenic species constitute the main bottleneck in the bivalve production process during the early stages of development, leading to high mortality rates and the rapid loss of production batches (Dubert et al., 2016b). Larval and spat mortalities associated to *Vibrio* spp. were described in hatcheries more than 50 years ago (Guillard, 1959) and still is awaiting a solution. Initially, Tubiash et al. (1965) proposed the term bacillary necrosis to describe a lethal disease of bivalve larvae and juveniles caused by bacteria classified as either *Aeromonas* sp. or *Vibrio* sp. Taxonomic affiliation of the etiological agents as *Vibrio* spp. was confirmed later (Tubiash et al., 1970). Subsequently, Elston (1999) suggested that the term bacillary necrosis should be replaced by a more descriptive name based on the type of infection. Hence, term vibriosis is generally extended to refer the bacterial disease in bivalve larvae and spat caused by pathogenic *Vibrio* species (Brown and Losee, 1978; Elston and Leibovitz, 1980; Elston, 1999). Different *Vibrio* species have been described as the etiological agent responsible of vibriosis promoting the larval and spat mortalities of different hatchery cultured bivalve species worldwide (Tubiash et al., 1965; Jeffries, 1982; Lodeiros et al., 1987; Nicolas et al., 1996; Estes et al., 2004; Gómez-León et al., 2005; Prado et al., 2005; Elston et al., 2008; Kesarcodi-Watson et al., 2009; Travers et al., 2014; Richards et al., 2014b; Rojas et al., 2015; Dubert et al., 2016d,e).

The aim of this review is to provide an overview on the vibriosis that affect bivalve larvae and spat in hatcheries due to its dramatic effects for the bivalve industry. We summarized the recent advances on this topic, with a focus on the aetiological agents, pathogenesis and preventive strategies described until now as well as the future prospects.

## Overview of the Pathogenic Vibrios for Bivalve Larvae and Spat: Species and Virulence Factors

One of the most important problems to define the current *Vibrio* species pathogenic to bivalve larvae and spat is related with their misleading taxonomic affiliation. In genomic era, techniques as multilocus sequence analysis (MLSA) or whole genome sequencing (WGS) are essential to provide a better understanding of the taxonomic position of the pathogenic *Vibrio* isolates and then to define them accurately (Sawabe et al., 2007, 2013; Urbanczyk et al., 2013). To avoid confusions, in the present review only the studies with *bona fide* identified strains were considered, but not those with presumptive *Vibrio* strains identified on the basis of phenotypic tests and not subjected to further molecular studies, i.e., studies by DiSalvo et al. (1978), Jeffries (1982), Lodeiros et al. (1987), Riquelme et al. (1995), Sainz et al. (1998), and among others.

**FIGURE 3:**
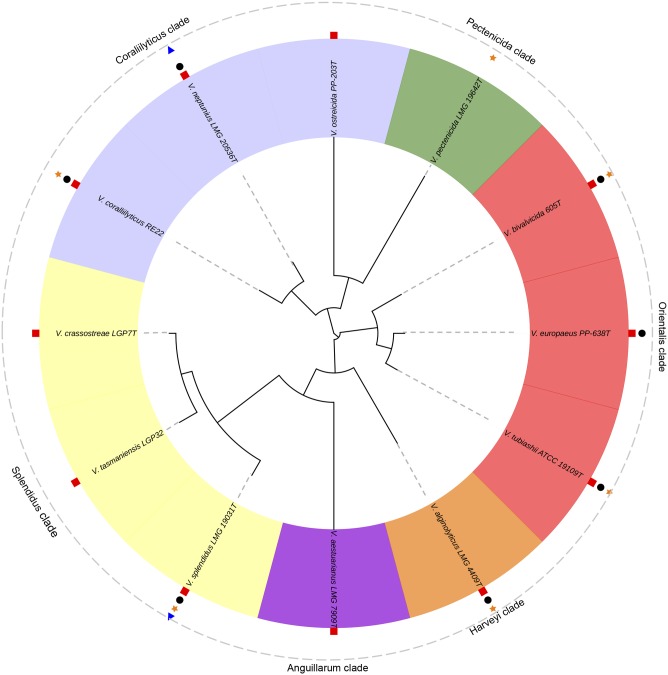
**Phylogenetic tree of the pathogenic *Vibrio* species responsible of outbreaks in bivalve hatcheries**. *V. tasmaniensis* and *V. crassostreae*, well-known pathogenic species for spat and juveniles in the natural environment, were also included. Analyses were based on the concatenation of the partial sequences of five housekeeping genes (*gyr*B, *fts*Z, *pyr*H, *rec*A and *rpo*A; 3073 bp) after multiple data alignment with MUSCLE software using ML algorithm (GTR+G model). Symbols show the pathogenicity of these species for larvae and/or spat of: oysters (red squares), clams (black circles), scallops (orange stars) and mussels (blue triangles).

In the next sections, the *Vibrio* species with importance for bivalve aquaculture due to the known pathogenicity for larvae and spat are summarized. We have also included the information available about their virulence factors. These species were clustered according with the *Vibrio* clades proposed by Sawabe et al. (2013) as easy way to stablish a good taxonomic approach (**Figure 3**).

### Anguillarum clade

#### Vibrio aestuarianus

*Vibrio aestuarianus* is an important pathogenic species responsible of massive mortalities of spat, juveniles and adult Pacific oyster (*C. gigas*) in France (Saulnier et al., 2010; Madec et al., 2014; Barbosa-Solomieu et al., 2015; Green et al., 2016; Azéma et al., 2016). Taxonomic studies let to classify these pathogenic French isolates as a new subespecies, *V. aestuarianus* subsp. *francensis* (Garnier et al., 2008), distinguishable from the American strains isolated in absence of animal mortalities (*V. aestuarianus* subsp. *aestuarianus*) (Tison and Seidler, 1983).

In relation with virulence factors, Labreuche et al. (2010) characterized from the extracellular products (ECPs) a zinc metalloprotease (Vam) with lethal effects for the host. Recently, Goudenège et al. (2015) have demonstrated that *varS* gene, which codes for a signal transduction histidine-protein kinase, is a key regulator of virulence and the secretion of Vam metalloprotease.

### Coralliilyticus clade

#### Vibrio coralliilyticus

This species was initially described as a coral pathogen responsible for coral bleaching (Ben-Haim et al., 2003a,b). Genard et al. (2013) described the physiological response of the *C. gigas* larvae during the bacterial infection of a strain of *V. coralliilyticus*, isolated from diseased oyster larvae and identified by 16S rRNA gene sequencing. Richards et al. (2014b) have demonstrated the virulence of the type strain ATCC BAA-450^T^ against Eastern (*Crassostrea virginica*) and Pacific (*C. gigas*) oysters (76.1–100% and 56.5–100% mortality, respectively). Recently, Mersni-Achour et al. (2015) demonstrated the high virulence of *V. coralliilyticus* against *C. gigas* larvae.

Sometimes, WGS is essential to elucidate the bacterial taxonomic position with accuracy. In fact, strains identified originally as *V. tubiashii* by partial sequencing of 16S rRNA gene (Estes et al., 2004; Elston et al., 2008) were further reclassified as *V. coralliilyticus* using such techniques (Wilson et al., 2013; Richards et al., 2014a). Hence, studies in which *V. coralliilyticus* is associated to outbreaks of vibriosis increased considerably (Tubiash et al., 1965; Estes et al., 2004; Elston et al., 2008; Gómez-León et al., 2008), extending its host range to other bivalve species as hard clam (*Mercenaria mercenaria*), flat oyster (*Ostrea edulis*), Atlantic bay scallop (*Argopecten irradians*) and naval shipworm (*Teredo navalis*). In summary, *V. coralliilyticus* constitutes serious threat for bivalve industry, being one of the most important emerging pathogens responsible of larval mortalities detected in shellfish hatcheries in France, New Zealand, and USA.

In relation with the virulence factors, several authors demonstrated that the high degree of virulence of some strains is associated to the production of high levels of extracellular metalloprotease (VtpA) and hemolysin (VthA) (Kothary et al., 2001; Delston et al., 2003; Hasegawa et al., 2008; Hasegawa and Häse, 2009a). Hasegawa and Häse (2009b) demonstrated that VtpA is a structural toxin to the host since this protein promotes significantly high toxicity to *C. gigas* larvae. Moreover, this metalloprotease appears to be the main secreted toxin in supernatants (Hasegawa et al., 2008; Hasegawa and Häse, 2009a). Later, Hasegawa et al. (2009) demonstrated that the VtpR protein, that belongs to the TetR family of transcriptional regulators, play a key role as a global regulator of potential virulence factors. Indeed, this protein activates VtpA production and the expression of an additional metalloprotease (VtpB). In addition, Spinard et al. (2015) discovered two other putative extracellular metalloproteases, one with similarities to the Epp protease in *V. anguillarum* and the other containing a conserved domain in the M4 family of metalloproteases. They also found different putative hemolysin/cytolysin genes, including a phospholipase/hemolysin with similarity to Plp in *V. anguillarum* and a hemolysin hlyA. Moreover, they identified a putative MARTX toxin operon encoding three transport proteins of the type I secretion system (T1SS). On the other hand, Weynberg et al. (2015) demonstrated by *in silico* comparative genomic analysis that bacteriophage genomes encoding toxin genes are integrated in *V. coralliilyticus* genomes, suggesting that virulence is driven by prophages and other horizontally acquired elements.

#### Vibrio neptunius

The first description of this species as a bivalve pathogen was reported by Prado et al. (2005) in a study with diseased flat oyster larvae. The strains, identified by 16S rRNA sequencing, showed a high degree of virulence against *O. edulis* and Manila clam (*Ruditapes philliphinarum*) larvae, with mortalities higher than 98% at 48 h (Prado et al., 2005; Dubert et al., 2016b). Kesarcodi-Watson et al. (2009) reported high mortality rates in Greenshell mussel larvae (*Perna canaliculus*) (100% in 2–3 days) during *in vivo* assays with other strain identified as *V. coralliilyticus/neptunius-like*. Later, these authors included Pacific oysters as susceptible host (Kesarcodi-Watson et al., 2012). However, the high degree of relatedness with *V. coralliilyticus* (Thompson et al., 2003, 2005) and the lack of genomic information hinder the accurate taxonomic affiliation of these strains as *V. neptunius*. Hence, comparative studies based on WGS should be done between *V. neptunius* and *V. coralliilyticus*.

#### Vibrio ostreicida

This species was described by Prado et al. (2014b). Type strain PP-203^T^ was obtained from inner surfaces of nursery containers with continuous mortalities of young *O. edulis* spat (Prado et al., 2005). Another two similar strains, PP-200 and PP-204, were obtained from different bins during the same outbreak and included in the taxonomic description reported by Prado et al. (2014b). These authors suggested the possibility that these strains were able to survive the water changes forming biofilms on the inner tank surfaces. In virulence assays, the type strain caused 86.4–98.5% mortality of *O. edulis* larvae after 24–48 h. No further reports of this species associated to bivalve mortalities or about virulence have been published. Further analyses based on WGS are needed to elucidate its taxonomic position within the clade.

### Harveyi clade

#### Vibrio alginolyticus

The first description of *V. alginolyticus* as a bivalve pathogen was reported by Tubiash et al. (1965). Indeed, they demonstrated its virulence for larvae of *M. mercenaria*, *O. edulis*, *A. irradians* and *T. navalis*. Luna-González et al. (2002) reported that scallops (*Argopecten ventricosus* and *Nodipecten subnodosus*) were more susceptible to the pathogen than other species tested, including *Atrina maura* and *C. gigas*, whereas Gómez-León et al. (2005) demonstrated the virulence of *V. alginolyticus* for carpet shell clam (*Ruditapes decussatus*) larvae and spat. On the other hand, Estes et al. (2004) associated the degree of virulence, at least for *C. gigas*, with an increase in the water temperature. In any case, isolation of this species has been not common in bivalve hatcheries in comparison with bivalve adults (Wang et al., 2016).

Recently, Castillo et al. (2015) identified putative virulence factors involved in adhesion and destruction of tissues (collagenases, arylsulfatases, proteases, and hemolysin), ABC-type transport systems (spermidine, putrescine, iron), and toxins (RTX, YafQ) from the draft genomes of two *V. alginolyticus* strains.

### Orientalis clade

#### Vibrio bivalvicida

*Vibrio bivalvicida* was described by Dubert et al. (2016d) who isolated three strains obtained from cultures of carpet shell clam in a Spanish hatchery. These strains led to high mortality rates (>96%) at 72 h in all experimental challenges including larvae of different clam species, such as *R. decussatus*, *R. philippinarum*, or *Donax trunculus*, as well as flat oyster. Recently, Rojas et al. (2016) reported the first isolation of *V. tubiashii* in Chile, demonstrating its pathogenic activity on the Chilean scallop larvae (*A. purpuratus*). However, the strain studied was really a representative of *V. bivalvicida* (unpusblished results) by means of WGS comparisons. Hence, it represents the first isolation of this species out of Europe. Overall, these results support the pathogenic potential of *V. bivalvicida* to kill the larvae of a broad range of bivalve species in the Atlantic and the Pacific oceans.

Interestingly, Dubert et al. (2016d) identified from the genome sequencing of the type strain three putative extracellular proteins characterized in other pathogenic *Vibrio* spp. A phospholipase/hemolysin and a HlyA hemolysin showing similarity with Plp and Vah1 of *V. anguillarum*, respectively, and a metalloprotease that shows similarity to VtpA of *V. coralliilyticus*. In addition, they also identified other five hemolysins, two phospholipases and type III (T3SS) and VI (T6SS) secretion systems involved in the extracellular secretion of effectors into a host cell.

#### Vibrio europaeus

*Vibrio europaeus* is the pathogen of bivalve larvae most recently described (Dubert et al., 2016e). Initially, this taxon was described as a subespecies of *V. tubiashii*, *V. tubiashii* subsp. *europaeus* by Prado et al. (2015). However, this study showed some taxonomic incongruities. Later, studies carried out by Dubert et al. (2016e) employing a polyphasic approach that included the WGS analysis, as well as phenotypic tests and chemotaxonomic techniques, supported their elevation to the rank of species. Strains described in the initial study were isolated from *O. edulis* and *R. philippinarum* during disease outbreaks in different Spanish hatcheries (Prado et al., 2005, 2015). Interestingly, Dubert et al. (2016e) included in the description of the new taxa a French isolate initially identified as *V. tubiashii*, and highly pathogenic for *C. gigas* larvae and spat (Mersni-Achour et al., 2014, 2015; Travers et al., 2014). Recently, Dubert et al. (2017) have reported a mortality event in larvae of other clam species, namely *R. decussatus*, involving this bacterial species. In summary, *V. europaeus* is an emergent bivalve pathogen responsible of severe losses that affected Spanish and French hatcheries (Travers et al., 2014; Prado et al., 2015; Dubert et al., 2016e, 2017)

In relation to the virulence factors, Mersni-Achour et al. (2014) identified an extracellular zinc metalloprotease belonging to the thermolysin family, close to predicted extracellular zinc metalloproteases of *V. tubiashii*. Later, Mersni-Achour et al. (2015) studied the two major fractions (F1 and F2) from ECPs by GP-HPLC and found differences in their toxicity to larvae (43% mortality for F1 fraction, 70% in the presence of the F2 fraction and 100% mixing both fractions). MS-MS analysis revealed a diversity of outer-membrane proteins in F1 (porin-like protein H precursor, outer-membrane channel protein, long-chain fatty acid transport protein, outer-membrane protein N and hypothetical proteins), whereas F2 showed a unique extracellular zinc metalloprotease. Recently, Spinard et al. (2016) found in the genome of the type strain PP-638^T^ two putative metalloproteases with 75% and 71% similarities respect to VtpA of *V. coralliilyticus* and to Epp of *V anguillarum*, respectively. Moreover, they detected three putative hemolysins and phospholipases encoded in the genome. Finally, T3SS and T6SS genes were also found. Interestingly, T6SS structural components are encoded on the p251-like megaplasmid, whereas the protein VgrG responsible for forming the puncturing tip of the T6SS appears to be encoded by two genes located in chromosomes 1 (Chr1) and 2 (Chr2) respectively.

#### Vibrio tubiashii

Strains ATCC 19109^T^ (=Mildford 74 J) and ATCC 10106 (=Mildford 27 O) were originally isolated in a North American hatchery from diseased juvenile clams (*M. mercenaria*) and oyster (*C. virginica*) larvae, respectively (Tubiash et al., 1965). Later, Hada et al. (1984) would describe the species formally. Pathogenicity of these strains was demonstrated in that report using larvae of clam (*M. mercenaria*), oyster (*O. edulis*), scallop (*A. irradians*) and shipworm (*T. navalis*). Takahashi et al. (2000) demonstrated a high degree of virulence degree of the strain ATCC 10106 using larvae of *C. gigas* in *in vivo* assays. Contradictory results were obtained by Richards et al. (2014b), since they did not detect significant mortalities for *C. gigas* larvae, but certain virulence (55.6–70.7% mortality) for *C. virginica* larvae. Scarcity of studies related with the virulence factors for *bona fide V. tubiashii* is due to the recently reclassification of some strains as *V. coralliilyticus*.

### Pectenecida clade

#### Vibrio pectenecida

First, Nicolas et al. (1996) isolated different strains associated to bacterial problems in hatchery cultures of great scallop (*Pecten maximus*) affected by recurrent larval mortalities. Among isolates tested, strain A-365 promoted considerable mortalities in *P. maximus* larvae, reaching 100% after 4 days. However, this strain did not cause significant losses in *C. gigas* challenges (>30% at 6 days). Later, Lambert et al. (1998) described the species taxonomically, proposing this isolate as type strain. This species has the particularity that does not grow on TCBS medium. Pathogenicity of the strain *V. pectenicida* A496 for *P. maximus* was demonstrated by Sandlund et al. (2006).

In relation to virulence factors, Lambert et al. (2001) demonstrated the toxic activity of *V. pectenicida* cytoplasmic extract on *P. maximus* hemocytes due to the vibrio hemocyte-killer toxin (VHKT).

### Splendidus clade

#### Vibrio crassostreae

This species was described as pathogenic for the spat of Pacific oyster in French farming areas, although no hatchery outbreaks have been reported yet (Faury et al., 2004; Gay et al., 2004a). Later, Lemire et al. (2014) demonstrated that *V. crassostreae* strains encode a putative outer membrane protein that is necessary for virulence. Recently, Bruto et al. (2016) demonstrated that this species is a non-virulent oyster colonizer that subsequently turns into a pathogen by acquisition of a virulence plasmid, essential for killing.

#### Vibrio splendidus

The high homogeneity of 16S rRNA gene within the Splendidus clade has led to difficulties in the taxonomy and identification of the strains and many authors have described closely related bivalve pathogens as *V. splendidus*-related strains. This group of strains has been commonly isolated from disease outbreaks. Indeed, pathogenicity of *V. splendidus*-related strains has been demonstrated for larvae of mussels (*Mytilus edulis* and *Perna canaliculus*), clams (*R. decussatus*), scallops (*P. maximus*), as well as for oysters (*C. gigas*) spat (Nicolas et al., 1996; Gatesoupe et al., 1999; Gay et al., 2004b; Gómez-León et al., 2005; Torkildsen et al., 2005; Sandlund et al., 2006; Kesarcodi-Watson et al., 2009; Rojas et al., 2015; Ben Cheikh et al., 2016; De Rijcke et al., 2016). Recently, Pérez-Cataluña et al. (2016) suggested a synonymy among *V. splendidus* and the later described species *V. hemicentroti*.

Knowledge of the virulence factors is essential to distinguish the virulent and non-virulent strains, since this species is commonly detected even in the absence of disease. A lot of information has been published on the virulence factors of *V. splendidus* strain LGP32 (see below). However, this strain was re-classified as *V. tasmaniensis* (Sawabe et al., 2013). Regarding virulence factors of *V. splendidus*-related strains, Macpherson et al. (2012) identified from the pathogenic strain DMC-1 a new haemolysin, termed vibrioaerolysin, with homology to aerolysin produced by several *Aeromonas* spp. Expression of the vibrioaerolysin is controlled by a ToxR-like gene located close to vibrioaerolysin gene, since the transposon insertion into the ORF of ToxR-like gene rendered mutants unable to produce haemolysin. Well known virulence factors, as Vsm and OmpU, have been also identified in the scallop pathogen *V. splendidus*-related strain JZ6 (Liu et al., 2013). For this strain hemolysis was temperature-dependent with highest hemolytic level at 10 °C and decreasing with the increase of temperature. Recently, Liu et al. (2016) carried out a comparative transcriptome analysis of *V. splendidus* JZ6, which shows highest virulence at 10°C. They identified 10 pivotal genes related to the virulence at 10°C involved in adhesion, protein secretion and virulence of *V. splendidus*: two genes (*secE* and *ftsY*) in Sec dependent pathway, two genes (flhG and VS_2437) for Flp pilus assembly and six genes (*toxS*, *cqsA*, *cqsS*, *rpoS*, *hapR*, and *vsm*) in “Vibrio Cholerae pathogenic cycle”. Moreover, a novel mono-ADP-ribosyltransferase (MART) toxin, named Vis toxin, produced by the oyster pathogenic *V. splendidus* 12B01 was characterized by Ravulapalli et al. (2015).

#### Vibrio tasmaniensis

Strain LGP32 (Gay et al., 2004a,b), formerly designed as *V. splendidus*, was taxonomically reclassified as *V. tasmaniensis* (Sawabe et al., 2013). This strain is a well-known pathogen for spat of *C. gigas* oyster in French farming areas (Gay et al., 2004a,b; Green et al., 2016). However, as for *V. crassostreae*, any outbreak of vibriosis due to this species has been reported in a bivalve hatchery until present.

Virulence factors of strain LGP32 have been widely studied. First, Vsm metalloprotease was identified due to a role in ECP toxicity for oysters. However, expression of *vsm* gene is not necessary for bacterial virulence in the oyster infection model when bacteria are injected (Le Roux et al., 2007). By means of comparative genomics, Binesse et al. (2008) demonstrated that Vsm was the major factor in the toxicity of the ECPs and that non-virulent strains lacked this marker. Complete genome sequence of strain LGP32 revealed homologues of genes usually associated with virulence, e.g., haemolysins, siderophore transport and utilization and adhesins (Le Roux et al., 2009). Interestingly, they found in Chr2 a haemolysin-co-regulated protein gene (*hcp*) and the *vas* operon, which encodes a type VI secretion system. Further studies revealed the importance of an outer membrane protein, OmpU porin, as the major determinant of *V. tasmaniensis* LGP32 pathogenicity in oyster experimental infections, contributing to resistance to antimicrobial peptides/proteins (AMPs) which are involved in *C. gigas* immunity, and confering adhesive properties (Duperthuy et al., 2010). These authors also elucidate the role of OmpU, as an adhesin/invasin required for β-integrin recognition and to attach and invade oyster hemocytes (Duperthuy et al., 2011), and defined this species as a facultative intracellular pathogen that manipulates host defense mechanisms to enter and survive in host immune cells.

## Recent Advances on the Pathogenesis of Vibriosis

In most reports is not clear if pathogenic vibrios are the primary causative agent, secondary opportunistic colonizers or commensals, due to the limitation of the experimental procedures used. Interestingly, the vast majority virulence assays in larvae were carried out by immersion in *Vibrio*-inoculated seawater being a good reflect of the natural route of infection (Tubiash et al., 1965; Lodeiros et al., 1987; Gómez-León et al., 2005; Prado et al., 2005, 2015; Kesarcodi-Watson et al., 2009; Richards et al., 2014b; Rojas et al., 2015, 2016; Dubert et al., 2016d,e). Obviously, time-course of mortalities depends on the degree of virulence of the pathogenic strain and the bacterial concentration inoculated. In contrast, spat and adults have shown reliance on infection via injection discarding the immersion for these assays (Duperthuy et al., 2011). However, in both cases, researchers generally inoculate a single *Vibrio* strain, whereas in the natural environment hosts are colonized by an assemblage of diverse vibrios (Gay et al., 2004a; Wendling et al., 2014; Le Roux et al., 2016). Thus, some studies have recently investigated the fact that the functional unit of pathogenesis is a bacterial clone, a population or a consortium. Interestingly, adaptive responses of the host to sympatric isolates have to be taken into account during the infection, in contrast to the virulence of the allopatric strains. For instance, cross-infection experiments in larvae demonstrated that *C. gigas* larvae showed lower mortalities with sympatric *Vibrio* combinations, demonstrating the adaptive potential of the host (Moehler et al., 2011; Wendling and Wegner, 2015).

Different reports have studied the mechanism of bivalve host response during vibriosis outbreaks by means of transcriptomic approach (Genard et al., 2013; Bassim et al., 2014). However, studies related with the onset and advance of the pathogenic bacteria are scarce in bivalves. Hence, we have described the new insights on this topic focused on the following of the infection process in bivalve larvae and spat from a bacterial point view.

### Pathogenesis in Bivalve Larvae

Clinical signs of vibriosis are well known in bivalve larvae in comparison with the processes of bacterial colonization and infection. In any case, the order of appearance of the clinical signs is the same regardless of the pathogenic *Vibrio* species: prodromal signs are constituted by reduction of larval motility, abnormal circular pattern of swimming and tendency to the quiescence by the inability to swim. At the peak of infection, dead and moribund larvae exhibit bacteria swarming on the margins and inside, disruption and/or extension of the velum, detachment of portions of the velum with cilliary action after all other soft tissues are destroyed. A phenomenon called ‘spotting’ is regularly observed, consisting in the accumulation and agglutination of moribund and dead larvae at the bottom of the tank (Tubiash et al., 1965; Elston, 1999; Estes et al., 2004; Prado et al., 2005, 2015; Torkildsen et al., 2005; Elston et al., 2008; Gómez-León et al., 2008; Kesarcodi-Watson et al., 2009; Beaz-Hidalgo et al., 2010; Mersni-Achour et al., 2015; Rojas et al., 2015, 2016; Dubert et al., 2016a,b).

**FIGURE 4:**
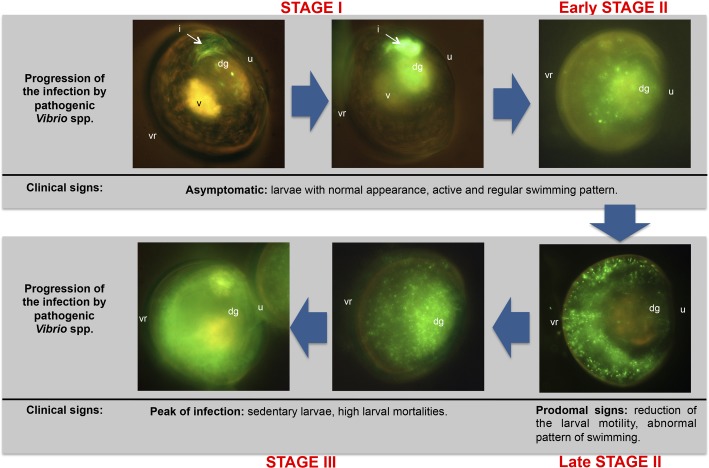
**Progression of the vibriosis infecting *R. philliphinarum* larvae with GFP-tagged pathogenic vibrios**. dg: digestive gland; i, intestine; u: umbo; v: vellum; vr: ventral region. Adapted from Dubert et al. (2016b).

First references about colonization routes for different bivalve species were exclusively described by means of histological observations (Tubiash et al., 1965; Elston and Leibovitz, 1980). However, microscopy techniques used in both studies did not let to distinguish the primary pathogen from the regular microbiota in the different larval tissues. Dubert et al. (2016b) described for the first time in bivalve larvae the complete colonization process by means of fluorescent tagging of pathogenic *Vibrio* species (*V. europaeus*, *V. bivalvicida* and *V. neptunius*), demonstrating that a bacterial clone is the most accurate approach for these bacterial species. Hence, they demonstrated the onset and advance of the vibriosis and accurately described the route of infection. Mortality and morbidity was studied in detail in that work using *R. philliphinarum* larvae as animal model and defined three infection stages (I, II and III) (**Figure 4**). In the Stage I, pathogenic *Vibrio* were filtered by the bivalve larvae through the vellum and entered the digestive system through the oesophagus and stomach, colonizing the digestive gland and quickly proliferating in the intestine during the first hours of infection. Stage II was characterized by the rapid expansion of the GFP-tagged *Vibrio* spp. to the surrounding organs in the body cavity from the dorsal to ventral region. From late Stage II, typical prodomal signs were observed. In the Stage III, pathogenic *Vibrio* spp. colonized completely the larvae at the peak of infection and the clinical signs corresponding to advanced infection were then observed. Interestingly, these authors demonstrated that the vibriosis is asymptomatic in the bivalve larvae during the early infection stages. This fact conditions the preventive treatments since once the pathogen is inside the larvae the infection process cannot be stopped.

### Pathogenesis in Bivalve Spat

Bivalve larvae are more susceptible to vibriosis than adults since the resistance to bacterial infection significantly increases with age of the bivalves (Gómez-León et al., 2008). Some authors have proposed different bacterial strategies to infect the bivalve host. Thus, the study of diseases using specific-pathogen-free (SPF) oysters has enabled the assessment of the infection process under natural conditions (Le Roux et al., 2016). Lemire et al. (2014) demonstrated that ecological populations often represent the functional unit of pathogenesis in which the presence of non-virulent strains increased the virulence of *V. crassostreae.* They suggested that non-pathogenic population could promote a high bacterial load necessary to either overcome host defenses or to induce expression of virulence factors via *quorum sensing* (QS). More recently, Bruto et al. (2016) identified abundant number of strains belonging to this species in oyster tissues which are nearly absent in the surrounding water. They identified virulent and non-virulent *V. crassostreae* strains and proposed the dynamic colonization of the host in which the non-virulent strains turn into a pathogen by introgression of a virulence plasmid. These authors hypothesized that the acquisition of the virulent plasmid is favored by the elevated host density in farming areas. On the other hand, Saulnier et al. (2010) demonstrated the synergistic effect over the virulence by means of co-infection of *C. gigas* spat with *V. tasmaniensis* and *V. aestuarianus*. Moreover, Goudenège et al. (2015) proposed that the functional unit of *V. aestuarianus* pathogenesis are clones. They clustered the virulent strains into two lineages and demonstrated that a regulatory gene *VarS* is essential for the infection.

## Preventive Treatments: Present and Future Prospects

The classical treatments were directed toward the complete elimination of bacteria from seawater, which constitutes an unfeasible and undesirable objective, because the cultures are not axenic and some bacteria even enhance larval development. At present, the efforts should be devoted to the microbiological improvement through the proper management of seawater circuit, the phytoplankton production systems, or the broodstock conditioning to maintain the necessary balance in the microbiota that allow the successful development of molluscan larvae.

### Water Treatments

In hatcheries, the water is subjected to treatments, including filtration, pasteurization, ozone, and UV radiation, with the aim to reduce the associated bacterial population. Generally, the first step is the decantation of the water pumped from the sea and, once most of the solid particles are eliminated, the water is treated. Filtration is an expensive treatment and only larval cultures and small-scale phytoplankton cultures receive maximum filtration water. Filtration is an advisable practice to reduce contents of bacteria as well as organic matter, and its results are better than the obtained by other systems like pasteurization, as showed by Lewis et al. (1988) in a hatchery of the Pacific oyster, *C. gigas*. The disinfection with chlorine or ozonization are alternatives also employed. Use of chlorine has shown some problems, including the interference in the larval mechanism of pumping (Vasconcelos and Lee, 1972) or the reactivity with organic nitrogen in the water that can produce toxic residues for marine organisms (Jorquera et al., 2002). On the other hand, application of ozonization can be complex and costly to disinfect aquaculture systems (Summerfelt, 2003) and can led to the appearance of oxidants toxic to aquaculture species (Richardson et al., 1982; Summerfelt, 2003). Although radiation of seawater with ultraviolet light has an unquestionable lethal power on bacteria, there are disagreements about the true effects when the treatment is used on water culture in hatcheries. Some authors have pointed out the advantages of this procedure, like the decrease of different bacterial populations including bivalve pathogens (Vasconcelos and Lee, 1972; Lodeiros et al., 1987), while other authors found important variations in effectiveness among samples and/or bacterial pathogens (Murchelano et al., 1975; Brown, 1981). Thus, the effects of UV-radiation treatment are variable and this variability may rise with factors such as the dose and the individual efficiency of the radiation unit, the water flow, or the presence of organic matter in the water (Brown and Russo, 1979; Liltved et al., 1995; Liltved and Cripps, 1999), existing the risk of a change from bactericide to only bacteriostatic effects. Therefore, the selection of undesirable populations resistant to the treatment, the high economic cost and the impossibility for the treatment of big volumes of water are the main disadvantages of this method.

In order to stabilize and increase larval survival, the development of new technologies and they integration into commercial hatcheries must be encouraged. The technology used in bivalve hatcheries has not progressed very much since the birth of this industry and still essentially relies on the static water methods developed in the 1960s for *C. virginica* and *O. edulis* (Loosanoff and Davis, 1963; Walne, 1974), which have been adapted to most cultured bivalves though without any great changes (Helm and Bourne, 2004). Nevertheless, about fifteen years ago, flow-through systems were developed for rearing bivalve larvae (Magnesen et al., 2006; Rico-Villa et al., 2008, 2009), some of them coupled with monitoring of different seawater parameters such as temperature, oxygen, pH, turbidity and ammonia, which allow the reduction in labor necessary for larval rearing and the increase of the larval density maintaining the same growth and survival rate but with high needs of water and energy.

Using recirculation systems (RAS) where water is treated and re-used, the seawater and energy needed can be reduced considerably. In addition, such systems would provide better water quality control, since water will be taken in only once per larval rearing cycle being not subject to fluctuations in the quality of the natural water supply. Furthermore, well-managed RAS has a stable microclimate that is hardly influenced by the low water intake. In finfish culture, this type of system has contributed to increased survival of the larvae in hatcheries and during later grow-out stages. Although at present there are no recirculation systems for bivalve larvae in commercial use, a number of studies have been performed on marine bivalve species (Widman, 1998; Pfeiffer and Rusch, 2000; Suantika et al., 2000; Xiongfei et al., 2005; Zohar et al., 2005) with promising results.

### Impact of the Antibiotic Use in Hatchery Environment

The use of antibiotics is one of the most widespread strategies for the control and prevention of vibrios in hatcheries. In hatcheries, antimicrobial agents have routinely been applied to water to treat and prevent disease, particularly during the first stages of bivalve development (Prado et al., 2014a; Dubert et al., 2016c). Therefore, antimicrobial agents as florfenicol, erythromycin, oxolinic acid and specially chloramphenicol are used in bivalve hatcheries to prevent the vibriosis and to improve the survival rates of larvae and juveniles (Lodeiros et al., 1987; Nicolas et al., 1996; Uriarte et al., 2001; Torkildsen et al., 2005; Campa-Córdova et al., 2006; Miranda et al., 2014; Dubert et al., 2016c). The use of chloramphenicol in Europe is currently banned in animals raised for human consumption, including aquaculture, because it has been associated with aplastic anemia and it is difficult to establish a safe level of human exposure (Schwarz et al., 2004). However, numerous studies have linked its use to higher larval survival rates and its efficacy in controlling the *Vibrio* populations and despite its prohibition, some authors justify that the brief use of chloramphenicol during larval development does not pose a risk to the consumer since the larvae are subsequently fattened in the sea for at least 1 or 2 years (Uriarte et al., 2001; Helm and Bourne, 2004; Torkildsen et al., 2005; Campa-Córdova et al., 2006).

The main risk associated with the extensive use of antibiotics is the development of resistant bacteria, which can transmit quickly resistance genes in the hatchery environment by horizontal transfer mechanisms (Zanetti et al., 2001; Kümmerer, 2004; Kitiyodom et al., 2010; Cabello et al., 2013; Miranda et al., 2013). In this way the use of antibiotics would have a detrimental effect in selecting resistant bacterial populations, including those with pathogenic potential. Indeed, Dubert et al. (2016c) suggested that these treatments limit the bacterial diversity and competition, favoring the proliferation of resistant vibrios in the hatchery environment and increasing the risk of bacterial contamination to the cultures. These authors demonstrated that the continued use of antibiotic (chloramphenicol) in a shellfish hatchery, far from optimizing and favoring the success of larval cultures, promoted the rapid development and persistence of resistant vibrios, most of them with pathogenic potential, that carry different R plasmids. Moreover, they demonstrated the transfer of R-plasmids from these resistant vibrios to other bacteria, including bivalve and human pathogens. This fact, constitutes a serious risk to the aquatic environment and public health. Interestingly, the persistence of these resistant populations in the hatchery environment could be promoted by subinhibitory and even residual concentrations of antibiotics (Beaber et al., 2004; Hastings et al., 2004; Buschmann et al., 2012; Davies and Davies, 2010; Andersson and Hughes, 2014).

Some authors have found the use of certain antibiotic in shellfish hatcheries promotes the co-selection of resistance to antibiotics (Dang et al., 2006; Dubert et al., 2016c). Hence, occurrence of multiple resistant genes in the same R-plasmid should be taken into account due to the mechanisms of co-resistance and cross- resistance (Courvalin and Trieu-Cuot, 2001).

The use of antibiotics in shellfish hatcheries is highly unadvisable since these facilities constitute a potential source of antibiotic residues and resistant bacteria to the aquatic environment. Even bivalve larvae and spat could act as delivery vehicles of resistant bacteria, including pathogenic vibrios, in different geographical locations and aquatic environments due to aquaculture exports. The exposure to these risks for aquatic environment and public health has to be taken into account.

### Eco-friendly Alternatives: Probiosis, Quorum Quenching (QQ), and Phage-therapy

In recent years, the use of probiotics become an interesting alternative to the utilization of antibiotics, although most of the studies about probiotics in aquaculture were focused on fish and crustaceans being scarce those focussed on molluscs. According to Verschuere et al. (2000), up to now the best adapted definition to application to larval cultures of bivalves, a probiotic would be a live microbial additive with a beneficial effect on the host, modifying the microbiota associated with the host or the environment, ensuring an optimal use of the feed or improving its nutritional value, improving the host response against the disease, or getting a better quality of its environment. In this sense, several studies have demonstrated high survival ratios when bivalve larvae are treated with probiotics prior to experimental infection with vibrios (Lodeiros et al., 1987; Douillet and Langdon, 1993, 1994; Gibson et al., 1998; Riquelme et al., 2000; Kesarcodi-Watson et al., 2012; Karim et al., 2013; Sohn et al., 2016a,b; Zhao et al., 2016). Among the antibiotic-producing marine bacteria used by these authors there are representatives of different bacterial taxa including, *Pseudoalteromonas haloplanktis* (formerly *Alteromonas haloplanktis*), *Aeromonas media*, *Alteromonas macleodii*, *Neptunomonas* sp., *Pseudoalteromonas* sp., *Pseudomonas* sp., *Vibrio* sp. or *Bacillus* sp.

In the last years, different isolates of *Phaeobacter gallaeciensis* (formerly *Roseobacter gallaeciensis*) and *P. inhibens* have received special attention by different research groups, due to their great spectrum of *in vitro* inhibition against pathogenic bacteria from aquaculture systems (Ruiz-Ponte et al., 1999; Prado et al., 2009, 2010; Kesarcodi-Watson et al., 2012; Sohn et al., 2016a,b; Zhao et al., 2016). Prado et al. (2009) in experiments performed in marine water, with phytoplankton cultures and with larvae of flat oyster (*O. edulis*) and clam (*R. philippinarum*) cultures confirmed its potential use as control method in mollusc hatcheries, if its action is allowed before the pathogens reach high concentrations in the system. Similar results were obtained by Kesarcodi-Watson et al. (2012) in challenge experiments of *P. maximus*, *O. edulis* and *C. gigas* larvae with different pathogenic vibrios including *V. coraliilyticus* and *V. splendidus*. On the other hand, Sohn et al. (2016a,b) and Zhao et al. (2016) demonstrated that probiotic of *P. inhibens* involves contributions from biofilm formation and antibiotic production and that, as for *P. gallaeciensis*, colonization in the system prior the introduction of the pathogens is needed for probiotic activity. Our research group has also obtained promising results on the improvement of larval survival and growth using a mixture of marine bacteria with probiotic activity in a scallop (*P. maximus*) hatchery (unpublished results).

More in depth works on probiosis in bivalve larval cultures are needed, to clarify the interaction among bacteria and the other live organisms, to establish the ability of probiotics to remain in the systems and to determine the appropriate dosage to achieve the highest effectiveness. In addition, methods to improve the conservation, storage and manipulation of probiotic in hatcheries are also needed.

One of the most promising alternatives to control pathogens in bivalve hatcheries is based on the inhibition of the expression of virulence genes, regulated in many aquaculture pathogens by bacterial cell-to-cell signaling, known as QS (de Kievit and Iglewski, 2000; Deep et al., 2011). QS is the regulation of gene expression in response to fluctuations in cell population density, which correlates with signaling molecule (autoinducer) concentration (Miller and Bassler, 2001; González and Marketon, 2003). The most thoroughly characterized Gram-negative, bacterial intraspecific autoinducers are *N*-acylhomoserine lactones (AHLs), which have been reported to accumulate in the culture medium, and bind to an AHL-receptor protein belonging to the LuxR family of transcriptional regulators. The activated LuxR/AHL complex then binds specific DNA sequences, resulting in the activation or repression of target genes, including in many cases the activation of important virulence phenotypes (Eberhard et al., 1991; Fuqua et al., 1994; Natrah et al., 2011). Some aquatic organisms, including micro-algae, macroalgae, invertebrates and also other bacteria, have the potential to disrupt QS by means of various different mechanisms (Natrah et al., 2011). A mechanism involves the production of compounds known as quorum sensing inhibitors (QSIs), that interfere with the detection of signal molecules (Givskov et al., 1996; Rasch et al., 2004; Teasdale et al., 2009). Such compounds were first described in the red marine algae *Delisea pulchra*, which synthesizes halogenated furanones with protective effect of both fish and shrimp from vibriosis (Givskov et al., 1996; Rasch et al., 2004). A second mechanism is the quorum quenching (QQ), which can be defined as the enzymatic inactivation of AHLs by the production of acylases or lactonases (Tait et al., 2005; Defoirdt et al., 2007; Romero et al., 2010, 2011). The use of AHL-degrading bacteria has been successful in increasing the survival of turbot (*Scophthalmus maximus*) and freshwater prawn have (*Macrobrachium rosenbergii*) larvae (Tinh et al., 2008; Nhan et al., 2010). Torres et al. (2013) detected and isolated AHL-degrading bacteria from a bivalve hatchery, including representatives of genera *Alteromonas* and *Thalassomonas* (further reclassified as belonging to genus *Thalassotalea*) (Deering et al., 2016), pointing out their potential to be employed to attenuate the production of virulence factors by bivalve pathogens. Since new agents for controlling bacterial diseases can be considered only when their efficacy is demonstrated using different challenge tests, further research is needed in order to elucidate the *in vivo* interactions of quorum-quenching microorganisms with aquaculture pathogens and animals.

Phage therapy also represents a promising alternative strategy for prevention of disease outbreaks (Housby and Mann, 2009). In natural environments, phages and their bacterial hosts maintain equilibrium. The “kill the winner” theory (Thingstad, 2000), and its further development “Cost of Resistance” (Våge et al., 2013), hypothesize that populations of bacteria that bloom are often controlled by phage infection, which subsequently reduces their numbers. Therefore, in natural ecosystems, wherever bacteria can be isolated, a specific phage can also generally be found (Chibani-Chennoufi et al., 2004; Stenholm et al., 2008).

Phages have been used for decades to effectively treat bacterial infectious diseases, including wound and gastrointestinal infections (Sulakvelidze et al., 2001; Sulakvelidze and Morris, 2001; Sulakvelidze and Kutter, 2005; Sulakvelidze, 2011) as well as to reduce food borne illnesses, including those caused by *V. parahaemolyticus* or *Salmonella* (Tan et al., 2014; Letchumanan et al., 2016; Pereira et al., 2016a,b). Commercial phages are now available for treating bacterial diseases in humans, animals (including aquaculture) and agricultural crops (Housby and Mann, 2009; Ly-Chatain, 2014). Thus, one Israeli company, Phage Biotech Ltd., has developed a phage treatment for *V. harveyi* in shrimp (Hodgson, 2013), the Australian biotechnology company Biologix is developing phage therapy for *Vibrio* sp. associated with mortalities in aquaculture (Letchumanan et al., 2016), and, in Baltimore, MD, Intralytix Inc. is also developing a phage treatment against *V. tubiashii* and related pathogens in larval oyster and clam hatcheries (Richards, 2014).

Phages typically are highly specific in terms of the bacterial species that they will infect and, indeed, commonly will only infect certain strains of any species (Housby and Mann, 2009). This host specificity of phages can be considered as both an advantage or disadvantage for phage therapy. Thus, although phages have a specific effect on the target bacterium and do not alter the normal microbiota of the culture system, a precise identification of the target bacterium is required before an appropriate phage can be selected for therapy (Housby and Mann, 2009). Another advantage of the use of phages is that they have the ability to disrupt bacterial biofilms (Azeredo and Sutherland, 2008). Thus, Luo et al. (2015) demonstrated that phages belonging to the *Siphoviridae* family successfully reduced *Vibrio* biofilms in an abalone farm. The potential emergence of phage-resistant bacteria or the role of some phages in the transfer of virulence genes are possible drawbacks of phage therapy.

More research would be needed in a near future to understand how the interaction of environmental factors, including pH, temperature, salinity and organic matter content, influences the efficiency of phage therapy in aquaculture systems. In this sense, Silva et al. (2014) demonstrated that salinity and organic matter although did not affect the survival of the bacteriophages, had a clear influence in the efficacy of the treatment. Therefore, to establish the best conditions to improve the efficiency of phage therapy and, in addition, to adapt its use in closed recirculated systems will be also key areas of research.

## Concluding Remarks

As considered along this review, the molluscan hatcheries contitute a singular and complex environment, i.e., “a sea of bacteria (mainly vibrios) in a world of larvae”, subjected to rapid changes that can lead to an imbalance in such ecosystem causing the culture failure and the death of the animals.

Despite the years passed since the first recognition of the role of the vibrios in as agents of larvae and spat diseases, the situation has not been solved yet. Hence, it is necessary in the coming years to focus the research on several aspects: (i) the *Vibrio* species implicated in the outbreaks. With the new genomic tools, *bona fide* identification of pathogens is feasible. New tools are necessary for early detection of these pathogens in the aquaculture systems. PCR procedures are available for some pathogens but procedures for use in the field are highly advisable; (ii) the virulence genes implicated specifically in the pathogenicity for bivalves should be determined in comparison with those already recognized for fish; (iii) knowledge on the disease onset. Are populations or clones the units of vibrio pathogenesis in bivalves?; and (iv) advances in the management of microbiological aspects of the water circulation system are devised. RAS systems specific for these invertebrates should be improved considering also the integration of preventive measures such as probiotic, QQ bacteria or bacteriophages, taking into account their desirable withstand period in these systems to successfully control the *Vibrio* populations.

## Author Contributions

JD, JB, and JR conceived and wrote, edited and approved the manuscript.

## Conflict of Interest Statement

The authors declare that the research was conducted in the absence of any commercial or financial relationships that could be construed as a potential conflict of interest.
